# The Development of a BMI-Guided Shape Morphing Technique and the Effects of an Individualized Figure Rating Scale on Self-Perception of Body Size

**DOI:** 10.3390/ejihpe10020043

**Published:** 2020-04-15

**Authors:** Geoffrey M. Hudson, Yao Lu, Xiaoke Zhang, James Hahn, Johannah E. Zabal, Finza Latif, John Philbeck

**Affiliations:** 1 Department of Health, Kinesiology and Sport, University of South Alabama, Mobile, AL 36695, USA; 2 Department of Computer Science, The George Washington University, Washington, DC 20052, USA; 3 Department of Statistics, The George Washington University, Washington, DC 20052, USA; 4 Department of Exercise and Nutrition Science, The George Washington University, Washington, DC 20052, USA; 5 Department of Psychology, The George Washington University, Washington, DC 20052, USA

**Keywords:** body image, self-perception, figure rating scale, avatar, virtual reality

## Abstract

The creation of personalized avatars that may be morphed to simulate realistic changes in body size is useful when studying self-perception of body size. One drawback is that these methods are resource intensive compared to rating scales that rely upon generalized drawings. Little is known about how body perception ratings compare across different methods, particularly across differing levels of personalized detail in visualizations. This knowledge is essential to inform future decisions about the appropriate tradeoff between personalized realism and resource availability. The current study aimed to determine the impact of varying degrees of personalized realism on self-perception of body size. We explored this topic in young adult women, using a generalized line drawing scale, as well as several types of personalized avatars, including 3D textured images presented in immersive virtual reality (VR). Body perception ratings using generalized line drawings were often higher than responses using individualized visualization methods. While the personalized details seemed to help with identification, there were few differences among the three conditions containing different amounts of individualized realism (e.g., photo-realistic texture). These results suggest that using scales based on personalized texture and limb dimensions are beneficial, although presentation in immersive VR may not be essential.

## 1. Introduction

Figure rating scales have been used to study body image issues for decades [1-3]; however, many studies using such scales acknowledge that the scales are not very realistic and that individuals may have difficulty relating to them [4]. This is particularly true of people that are not of European descent, as early figure rating scales only depicted Caucasian body types [4-6]. Because these figure rating scales evaluate how a person perceives themselves, having a realistic and culturally relevant rating scale is important for precise assessments [1,5,7]. More culturally specific scales have been created, specifically for African American female populations [8,9]. Pulvers et al. [5] also addressed this concern of cultural relevance by developing an instrument that was more ethnically neutral. Their specific figure rating scale was validated with an urban, African-American population, but the authors concluded that their instrument should be perceived well by a diverse range of ethnicities.

Going beyond cultural specificity, it has been hypothesized that an individualized figure rating scale (i.e., tailored to the individual’s actual body dimensions and surface features) could provide an even more precise evaluation of how a person perceives their body shape [3,10]. In both clinical and research settings, the precision of perceptual body image evaluation becomes relevant in illnesses where the key symptom is a disturbance in body image perception—e.g., eating disorders [11]. The purpose of the current study was not to investigate body image disturbances in a clinical population but, rather, to determine if utilizing realistic, individualized avatars would improve the accuracy with which a person could identify their image. As established by Cash and Deagle [12] and echoed by many other researchers [10,13-15], body image is a multi-component concept that incorporates numerous constructs. Of the two major domains of body image (perceptual and attitudinal) [12,13], the perceptual domain was of primary interest in the current study. Given that the interest is in the subject’s ability to estimate their body size based on an image of themselves, research from Longo et al. [15] might argue that these depictive methods lie somewhere in between the perceptual and attitudinal domains of body image. Nonetheless, the primary research question of the current project was to study the participants’ self-perception of changes in size of their own picture with varying degrees of realism). In this sense, the current study was modeled after one conducted by Altabe and Thompson [16]. These researchers utilized the Stunkard Figure Rating Scale [6] and asked subjects to identify the figure that reflected how they thought they look, the figure that reflected how they felt, and then their ideal figure. They then calculated the discrepancies in those question responses and correlated them with scores from three subscales of the Eating Disorders Inventory (EDI) [16,17]. The approach of the current study was similar, in that, behaviorally, our interest was in how women would use figure rating scale images to answer questions about their body self-perception. The novelty in the approach of this study is that instead of utilizing traditional figure rating scales, the researchers implemented an innovative approach to morphing a 3D image of the subject in order to generate individualized figure rating scales that varied in degrees of realism.

This leads to the primary research question of how realism in a figure rating scale would affect subjects’ responses to body self-perception questions. Unlike the study of Altabe and Thompson [16], the current study utilized the more culturally neutral scale created by images obtained from Pulvers et al. [5,18], where each image presented to participants was associated with a body mass index (BMI). This was termed the baseline condition, while the other three conditions used optically scanned images of the participant’s own body that were morphed to create visualizations of the participant’s body that differed in BMI. In a within-subject manner, participants in the current study used all four types of visualization to answer the following three questions: (1) Which figure best reflects how you think you look today? (2) Which figure is the size and shape that you would most like to be? (3) Which figure is the size and shape that you feel would be most realistic for you to maintain? Questions 2 and 3 were also used to determine if there was a relationship between desired body shape and how a participant’s body image affects their quality of life (QOL) [19].

A variety of earlier studies have used photographs or videos of the participants themselves, distorting the images to yield visualizations of different body shapes (for a review, see Mülbert et al., 2017 [10]). More recently, studies have utilized commercially available optical body scanners to generate realistic, individualized avatars of their study subjects that are digitally morphed to create different body shapes for evaluation [3,20,21]. In particular, several studies have directly manipulated whether or not avatars were individualized. These studies have shown, for example, that aspects of individualization impact women and men differently, with individualized body shape being more important for women and photo-realistic texture being more important for men [21]. At least in females, subjects were more accurate in using avatars to match their own body shape if the avatars were not individualized; if avatars were individualized, women of lower BMI tended to underestimate their body size and women of higher BMI tended to overestimate their body size [22]. Generating and presenting individualized avatars are resource intensive, and this limits its potential for use in both clinical and research settings. Many types of rating scales using generalized figures can be printed on paper and used to measure perception of body shape quickly and cheaply, with virtually no specialized equipment required. Thus, an important question that remains open is how to determine what is an acceptable tradeoff between maximizing realism and minimizing resource demands associated with generating and presenting highly individualized avatars. This would require extensive experimentation to address in a comprehensive way. To begin to address this issue, here, we assessed how body shape ratings vary across several levels of realism, spanning the range between an established generalized rating scale to highly individualized, 3D avatars. The importance of this endeavor was recently highlighted in a study by Cornelissen et al. [23]. These authors described an epidemiological study [24] where participants estimated BMI based on viewing images on a figure ratings scale. The authors concluded that the measurement error in a study of this nature would be reduced by improving the precision of these more realistic figure rating scales. As a result, this could theoretically lead to fewer misclassifications of people as overweight or obese when estimated from figure rating scales [23].

Consequently, an additional aim of the current study was to develop an innovative way to more realistically generate morphed 3D avatars, as methods previously used for generating individualized avatars lacked anatomical specificity. Simulating increases in BMI by optically stretching images horizontally or by inflating 3D models of a body similar to a balloon, as has been done in earlier studies [10], do not realistically capture how adipose tissue is deposited in the human body. To address this, the existing avatar-based self-perception studies [3,20,21,25,26] have taken a more sophisticated approach, mapping variations in BMI onto variations in shape using a statistical learning model. This data-driven approach builds upon work by Allen, Curless, and Popovic [27], which analyzes the variation of 3D body shapes in the scope of the body shape dataset using principal component analysis (PCA). Body shape descriptors derived from PCA can thus be linked to external attributes, such as weight, height, BMI, arm length, and inseam, using a linear regression model by solving a least square problem ***Y*** = ***βp***, where ***Y*** denotes the external attributes, ***β*** denotes the mapping coefficients, and ***p*** denotes the shape descriptors. By mapping the shape to the external attributes, BMI variations can be quantified with the variation of the shape descriptors, and vice versa. The linear regression model naturally predicts shape changes with varying BMI, but the model is limited by several key factors. First, the linear regression model inherently introduces high bias to the mapping between the shape descriptors and the external attributes due to the model simplicity. Therefore, the shape changes corresponding to BMI cannot be quantified accurately. Second, the linear regression model inherently simplifies the shape variation by averaging over multiple sources of shape variation to extract a more general pattern of how the shape varies with BMI. Third, generalizability is a concern for this data-dependent model because the shape descriptors are derived from the PCA over the training dataset. Therefore, data sparsity or population distribution bias in the training dataset will degrade the model generalizability. Part of the novelty of this pilot study was the development of a new technique for a BMI-guided shape morphing approach that avoids the stated limitations with the PCA approach to shape morphs.

Building upon previous work in women [21,22], and in keeping with our goal of exploring the tradeoffs between realism and practicality, here, we assessed the accuracy of body perception judgments across the following three levels of individualized realism: (1) silhouette condition: here, avatars were based on optical scans of the participant’s own body (thus preserving individualized body shape and limb dimensions) but without any texture. Thus, some individualized realism is present and represents the participant’s own body shape and size. (2) 2D-with-texture condition: this condition again involved optical scanning to create avatars of the participant’s own body, but this condition heightened realism by adding an individualized and photo-realistic surface texture. (3) 3D-with-texture condition: this condition heightened realism still further by using a three-dimensional, photo-realistic avatar of the participant’s own body, created using optical scanning, and seen in a 3D immersive visual display. These individualized avatars with the BMI-guided shape morphing approach were also compared to the baseline condition: a non-individualized figure rating scale consisting of a series of cartoon-like line drawings [5]. Our purpose was to determine the effect that these varying degrees of realism in the figure rating scale would have on the participants’ ability to identify the image of their size as well as the influence on the discrepancy between the real and ideal images and the relationship of how these findings relate to their QOL.

## 2. Materials and Methods

### 2.1. Participants

This pilot study consisted of eight female subjects with a mean age of 32.0 ± 12.2 years. Of these female subjects, five were Caucasian, one was African-American, one was Asian, and one was Hispanic. Their average BMI was 22.9 ± 4.3 kg·m^−2^, and average percent body fat was 28.7 ± 9.4%. The subjects were drawn from a cohort of 160 volunteers who previously had their 3D body shapes generated as part of a study designed to validate an optical 3D body scanning system [28]. This original study recruited both male and female participants that had to be between the ages of 18 and 55 and have a BMI between 18.5 and 40 kg·m^−2^. Given that the original study was validating an optical body scanning system, participants could not be pregnant, have a history of eating disorders, have deep facial beards (>0.5 in), or be missing a portion of a limb. BMI and percent body fat were determined in this previous session via dual-energy x-ray absorptiometry (DXA; Lunar iDXA Madison, WI), which was conducted approximately eleven months prior to the behavioral testing reported here. Of the 160 participants in this previous study, 86 were female and were contacted by e-mail and asked to return for a follow-up session where they would be asked a series of questions regarding their morphed 3D images. Given the time- and resource-intensive nature of generating and presenting individualized 3D avatars, this new preliminary study was limited to the eligible females from the original scanning validation study that expressed interest in participating in this follow-up study. On the day of behavioral testing, subjects self-reported their current weight, and there were no significant differences between the self-reported weights and the values previously measured. The methods for both studies were in accordance with the Declaration of Helsinki and approved by the Human Subject’s committee of the George Washington University Institutional Review Board. Participants provided written informed consent prior to participation in the original validation study as well as the follow-up study described in this manuscript. Participants in the current study chose to join this self-perception follow-up trial after completing the optical scanning validation study [28] and were informed that they could withdraw from this follow-up study at any time. However, no participants decided to withdraw once they started the current study.

### 2.2. Survey Instruments

As previously mentioned, the self-perception questions utilized in the current study were modeled after that from Altabe and Thompson [16]. The responses from Question 1 (Which figure best reflects how you think you look today?) provided a means of estimating the accuracy of participants’ perceived body shape (measured using the four image types) relative to their actual BMI. Unlike the Stunkard figure rating scale [6] utilized previously, we chose a more culturally relevant figure rating scale that structured the size of the different images based off of incremental increases in BMI [5,18].

For Question 2 (Which figure is the size and shape that you would most like to be?), we sought to determine the relationship between participants’ desired body size and their actual size. This question was similar to the “ideal figure” question of previous studies [16], which utilized it to evaluate body dissatisfaction. The current study further wanted to determine how the degrees of realism among the four image types would affect that relationship between desired and actual body size. Lastly, Question 3 (Which figure is the size and shape that you feel would be most realistic for you to maintain?) provided participants’ estimation of achievable weight and shape norms or self-efficacy in weight management.

Across the four image-type conditions, there is a general increase in individualized realism and a corresponding increase in the visual cues to body shape. One prediction was that these increases would drive judgments of current body shape (i.e., responses to Question 1) toward increasing accuracy relative to the physical BMI. Alternatively, effective cues to body shape may be available even in generalized line drawings, and increasing levels of individualized realism above and beyond this may not provide much additional benefit. Our study goes beyond the scope of previous studies [21,22], which found that image individualization negatively impacted body shape self-perception in women by assessing whether this holds true across visualizations differing more widely in realism.

The Body Image Quality of Life Inventory (BIQLI) [19] is a multidimensional measure designed to evaluate the impact of body image on various aspects of a person’s QOL. The BIQLI evolved to become a 19-item questionnaire evaluating QOL domains that were deemed to be impacted by body image. The 19 items are scored on a 7-point bipolar scale where participants rate how strong of a negative or positive impact (−3 to +3) their body image has on specific elements of their QOL [19]. The BIQLI has been shown to be reliable and valid in numerous populations (Cronbach’s alpha of 0.93–0.95 [19,29]), is internally consistent and stable for up to a three-week period (test–retest reliability of 0.79 [19]), and has even been translated and validated in numerous languages [19,29-32]. More specifically, it has demonstrated the ability to differentiate among clinical and non-clinical groups, particularly groups of college-aged women [33]. Given the nature of our research question and our sample of college-aged women, the BIQLI was an appropriate choice in this endeavor over the EDI [17] because we were not testing a clinical sample and our research interest was more in the relationship between desired body shape and how a participant’s body image affects QOL.

### 2.3. BMI-Guided Explicit Shape Morph

Three-dimensional body shapes were generated using an optical 3D body scanning system, which uses two Microsoft Kinect v2 sensors mounted on a stationary tripod as described previously by Lu et al. [28]. Previous methods have implicitly estimated body shape differences using a statistical model [3]. Although this method produces realistic-looking results, the accuracy cannot be easily validated. To remedy this, we simulated the shape differences based on a two-compartment (2C) body composition assumption, in which the target shape is directly derived from the BMI. To simulate body shape changes with varying BMI, we used an explicit shape morph algorithm based on a B-spline skinning deformation model. In this algorithm, the original 3D body shape of the subject is reconstructed by an optical 3D body scanning system [28]. We used the subject’s weight, height, and percent body fat as inputs for the BMI-guided shape morph simulation. Our goal was to derive different body shapes from the original body, varying in BMI. Under the 2C model, body composition can be classified into fat mass and fat-free mass [18]. The 3D spatial distribution of fat mass and fat-free mass can be estimated by analyzing the 3D body shape. The change of 3D body shape can be directly mapped to the change of fat mass and fat-free mass and thus to the change of BMI. We first parameterized the 3D body shape deformation and then simulated the 3D body shape morph with a target BMI by optimizing the deformation parameters. For this study, BMI was chosen as the best variable to manipulate the shape changes in our avatars in order to simulate changes in body weight. A primary reason for this was because the baseline condition for this study was comparing the individualized, photo-realistic avatars to the generalized figure rating scale from Pulver et al. [5], which also based changes in body size on changes in BMI. Secondarily, while body composition was built into the algorithm (Equation 1) used to calculate the volume of the newly generated morphed avatars, it is impossible to predict how body composition changes as a person increases or decreases their size. For example, as a person gets larger, they may be adding adipose tissue or muscle mass, while as their size decreases, they may be losing both muscle and adipose tissue mass. Although it may not always be the case, BMI and body volume have a stronger, more predictable relationship. Thus, BMI was chosen as the primary variable to drive the changes in volume of each morph, assuming that body composition was to remain constant.

For the 3D body shape deformation parametrization, we defined an abstract skeletal structure on the original 3D body shape and painted skinning weights associated with the bones, which we then iteratively blended with the vertex’s weights as well as its neighbors’ weights to smooth the weight transitions. We set up multiple key points along each bone to parameterize the surface mesh deformation. Each key point was associated with an in-plane scale factor to grow or shrink the surface on its planar direction orthogonal to the skeletal structure. In between the control points, the in-plane scale factors were interpolated using B-spline interpolation. In practice, to reduce the complexity of our deformation control and to enhance the flexibility of our deformation model, we separated the in-plane scale factors into a global scale factor and a set of on-bone influence controls. The global scale factor controls the overall body shape deviation from the original scanned surface, where the positive global scale corresponds to the surface growth, whereas the negative corresponds to the surface shrink. The on-bone influence controls define how sensitive the sub-region of the mesh is to the change of the global scale. This is customizable to realistically simulate how a real body would look based on a certain BMI. For each vertex on the mesh surface, we calculated its projection on each bone to retrieve the scale factors (global scale × B-spline interpolated on-bone influence control). We calculated the deformed vertex positions affected by each bone. Then, we created a weighted sum of the deformed vertex positions using the blended skinning weights to derive the final vertex position.

To simulate the shape morph with a specified BMI, we determined the fat mass *m_FM_* and fat-free mass *m_FFM_* of the subject using DXA. We estimated the morph target volume *V_target_* for the 3D body shape with Equation (1) corresponding to the given BMI target. We assumed the density of the fat component *ρ_FM_* to be 0.900 kg/L. The density of the fat-free component varies with gender and ethnicity. For non-Black females, we set the density *ρ_FFM_* to 1.100 kg/L according to the Siri Equation [34]. For Black females, we set the density *ρ_FFM_* to 1.106 kg/L according to the Ortiz Equation [35]. We optimized the deformation parameters to reach the target body volume *V_target_*.

(1) $$V_{target}=\frac{BMI\cdot Height^{2}-m_{FFM}}{\rho_{FM}}+\frac{m_{FFM}}{\rho_{FFM}}$$

**Figure 1.**
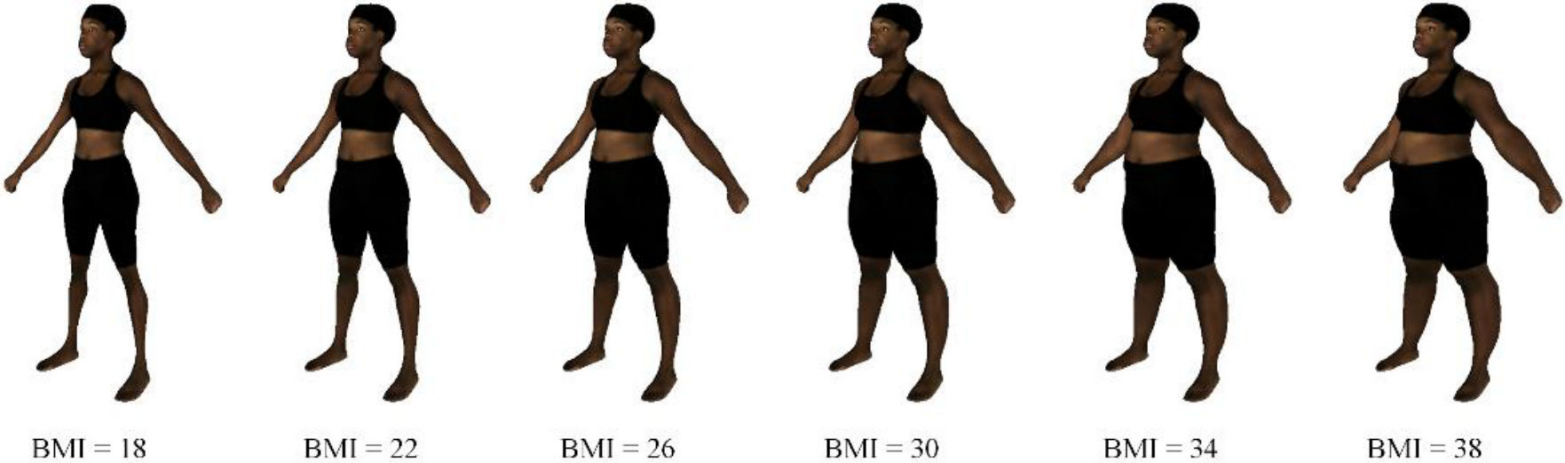
Shape morph simulation for different body mass index (BMI) values.

Figure 1 illustrates examples of the shape morph simulation with specified BMI targets. The examples show that our method can simulate realistic-looking body shape changes with various BMI settings.

### 2.4. Procedure

Subjects completed the BIQLI [19] prior to their arrival for the self-perception testing. To begin the self-perception testing, subjects were presented with four types of figure ratings scales, the order of which was assigned randomly so that no subjects viewed the different figure types in the same order. The four visualizations were as follows: (1) the Pulvers et al. [5] figure rating scale (black line drawings against a white background); (2) personalized silhouettes based on the subject’s optical scan, seen face-on; (3) textured, photo-realistic 2D figures based on the subject’s optical scan and seen face-on; and (4) textured, photo-realistic, virtual reality (VR) figures that could be rotated slowly to provide dynamic changes in viewing perspective. The first three visualizations were displayed on a flat computer monitor, while the VR figures were viewed in an Oculus Rift headset. This VR condition was also “immersive”, in that head motions were tracked and used to simulate the avatar as being at a stable location in the world relative to the subject’s perspective. Thus, head motions elicited some changes in perspective due to motion parallax. Within this VR environment, participants were also able to rotate the 3D image to view it at the 45-degree or canonical view, as this angle has demonstrated improved accuracy in discriminating among bodies of different sizes [13,23,26].

**Figure 2.**
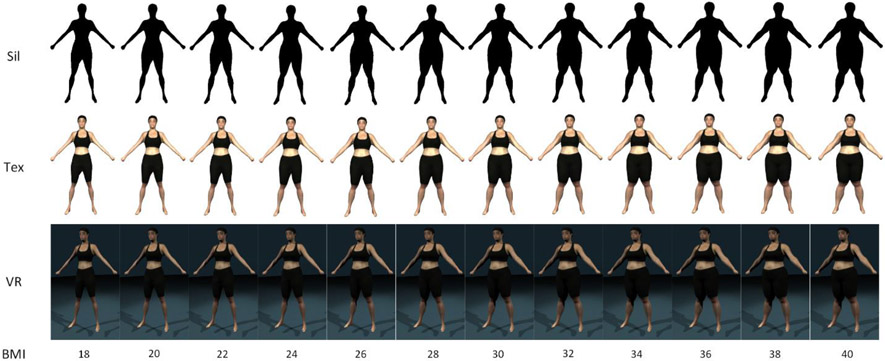
An example of the figure ratings scales of the silhouette (Sil), 2D texture (Tex), and 3D virtual reality (VR) conditions.

Figure 2 shows examples of the silhouette (Sil), 2D textured (Tex), and 3D VR visualizations. Each visualization contained 12 figures separated by increments of approximately 2 BMI (kg·m^−2^) with the lower and upper bounds of approximately 18 and 40 kg·m^−2^. Subjects were able to use the scroll wheel on a mouse to manually cycle between the different images (effectively increasing or decreasing the depicted BMI).

A potential limitation of previous studies is that the image depicting the participants’ actual BMI was always in the middle of the stimulus set, and there were an equal number of morphed images with smaller and larger BMI’s on either side. For example, Piryankova et al. [25] and Mölbert et al. [3] generated morphed images that were ±5–20% BMI of the true image. Based on the concepts of contraction bias [13,36], participants would be biased toward selecting images in the middle of the presented range, this could thus bias responding accuracy (given that the real image was always the middle image). To mitigate this and to provide symmetry among the four test conditions, the images were confined within the range of 18–40 kg·m^−2^ as they were in the Pulvers’ figure rating scale [5]. To further limit contraction bias, the starting body size (prior to adjustment) was randomly selected within the 18–40 kg·m^−2^ BMI range, instead of always starting at the smallest or largest image. Additionally, in our three avatar conditions, rather than presenting a series of static images differing in size, participants used the scroll wheel of a mouse to dynamically adjust the size of the avatar. Subjects were then asked to scroll through the different sized images and select an image to answer each of the three research questions: (1) Which figure best reflects how you think you look today?; (2) Which figure is the size and shape that you would most like to be?; and (3) Which figure is the size and shape that you feel would be most realistic for you to maintain? As was previously done [16,37], we calculated discrepancy scores of the real figure and their answers to Question 1, as well as between the real figure and answers to Question 2. The answers to these questions and the discrepancy scores were then correlated with different items from the BIQLI [3,19].

### 2.5. Statistical Analysis

We first compared the four types of figure rating scales to see if they would lead to significantly different BMI responses for the same question. We constructed a 95% confidence interval for each pairwise difference between image types. If zero was not contained in a confidence interval, then the BMI values of the corresponding two image types were considered to be significantly different at the significance level *α* = 0.05. Due to the small sample size (n = 8), we created each confidence interval using 199 bootstrap samples. The number of bootstrap samples was chosen to be relatively large but still less than the total number of samples of size 8 with replacement (256 = 2^8^). Due to the exploratory nature of the study and the small sample size, we forewent any corrections to the confidence levels of these multiple intervals (e.g., Bonferroni corrections).

We also constructed a 95% bootstrap confidence interval for the Pearson correlation between each BMI difference and subjects’ answers to each question of the BIQLI (integer ratings ranging from −3 to 3 [19]). MATLAB R2018b was used to perform all aforementioned statistical analyses.

## 3. Results

**Figure 3.**
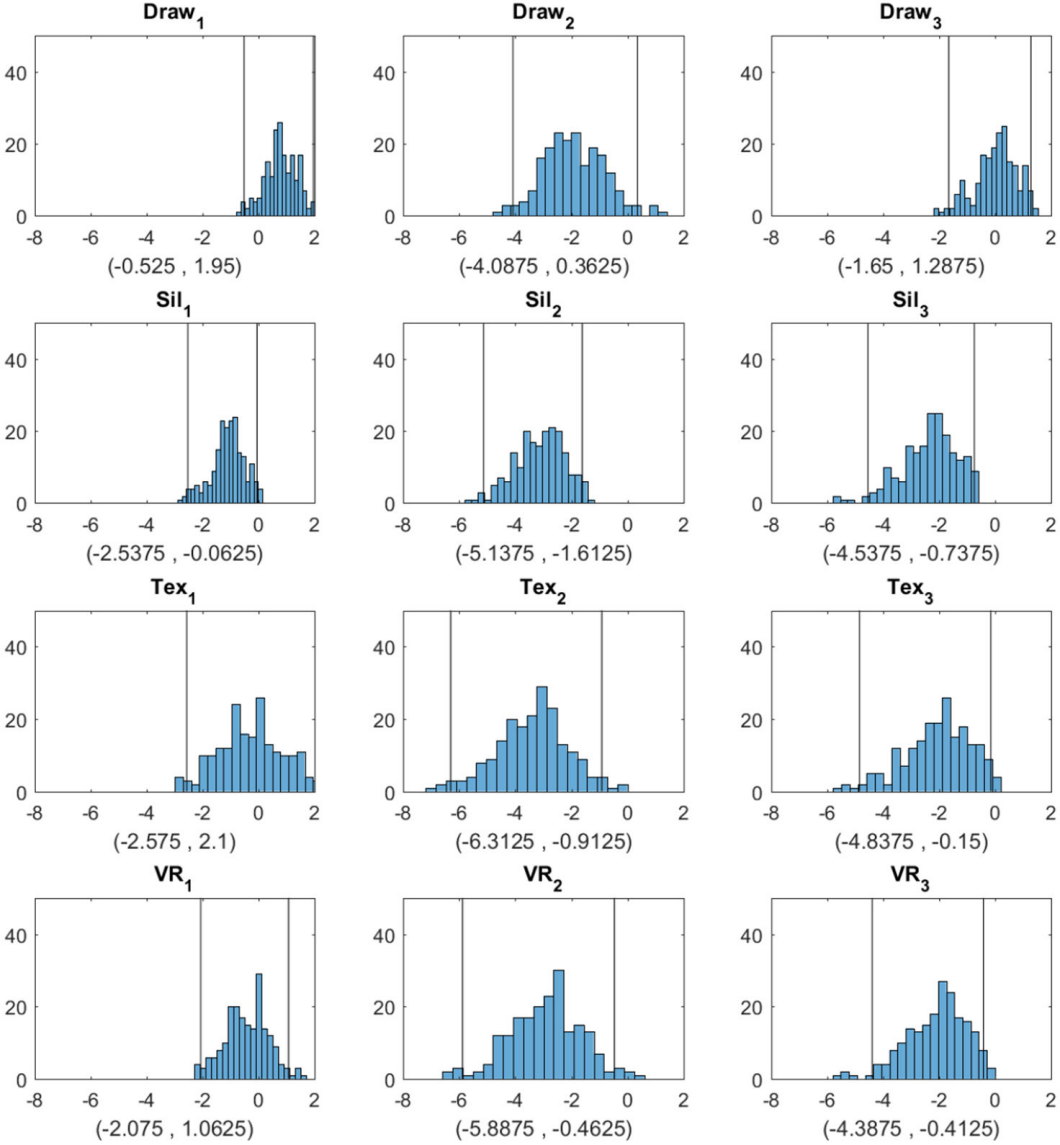
Histograms and 95% confidence intervals obtained by bootstrap for the difference between the BMI selected from each image type and the true BMI (Draw = hand drawing, Sil = silhouette, Tex = textured silhouette, and VR = virtual reality). The three columns correspond to Question 1 (Which figure best reflects how you think you look today?), Question 2 (Which figure is the size and shape that you would most like to be?), and Question 3 (Which figure is the size and shape that you feel would be most realistic for you to maintain?), respectively. The two vertical lines in each plot correspond to the lower and upper bounds of each confidence interval, respectively.

**Figure 4.**
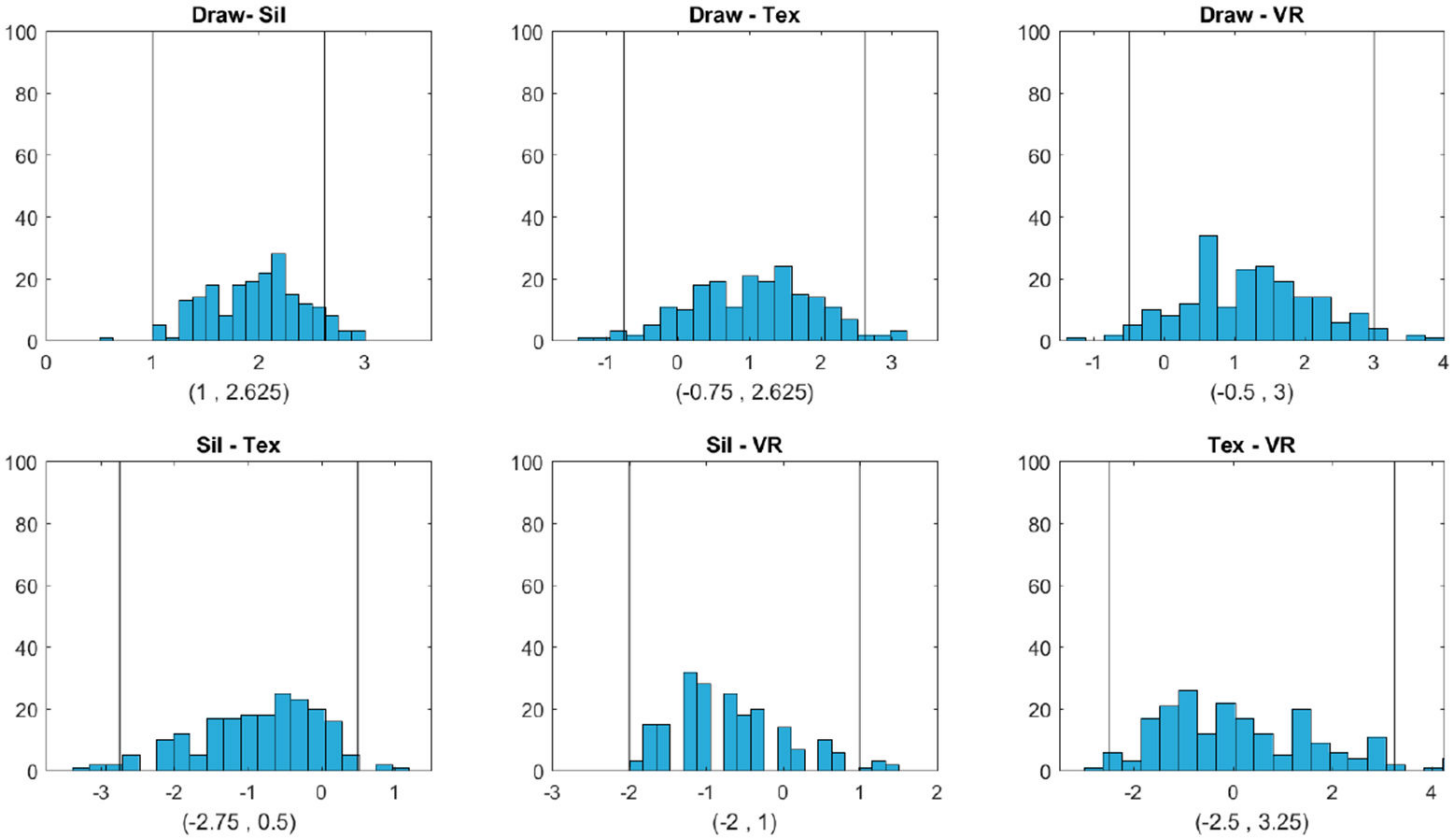
Question 1: Which figure best reflects how you think look today? Histograms and 95% bootstrap confidence intervals for the pairwise BMI differences between the four image types (Draw = hand drawing, Sil = silhouette, Tex = textured silhouette, and VR = virtual reality) for Question 1 (Which figure best reflects how you think you look today?). The two vertical lines in each plot correspond to the lower and upper bounds of each confidence interval, respectively.

The bootstrap distributions and confidence intervals are given in Figures 3 and 4. We assessed how closely the response BMI values matched subjects’ real BMI across the four image types. For each subject, we first computed the difference between the response BMI for each image type and the subject’s real BMI and then constructed a 95% confidence interval for this difference based on 199 bootstrap samples. The bootstrap distributions and confidence intervals are given in Figure 3.

Figure 3 shows that for Question 1 (Which figure best reflects how you think you look today?), the BMI values chosen by subjects were most accurately aligned with their actual body shape in the 2D textured and 3D VR conditions, as indicated by the zero point of the difference scores being approximately in the center of the distributions and well within the 95% confidence intervals. For the hand-drawn condition, response BMI values were somewhat overestimated relative to the physical BMI, although not significantly (the zero point fell within the 95% confidence interval, albeit near the lower boundary). For the silhouette condition, response BMI values significantly underestimated the physical BMI. For both Question 2 and Question 3, people tended to select significantly lower BMI values than their real ones for most image types. For Question 2, responses in the drawing condition did not differ significantly from zero, although there was a tendency toward lower BMI values, similar to the other conditions. Thus, there was a general tendency to desire a smaller body size than the current size. For Question 3, for the silhouette, 2D textured, and 3D VR conditions, subjects again chose significantly lower BMI values than their current BMI. Responses for the drawing condition, meanwhile, did not differ significantly from the current BMI.

**Figure 5.**
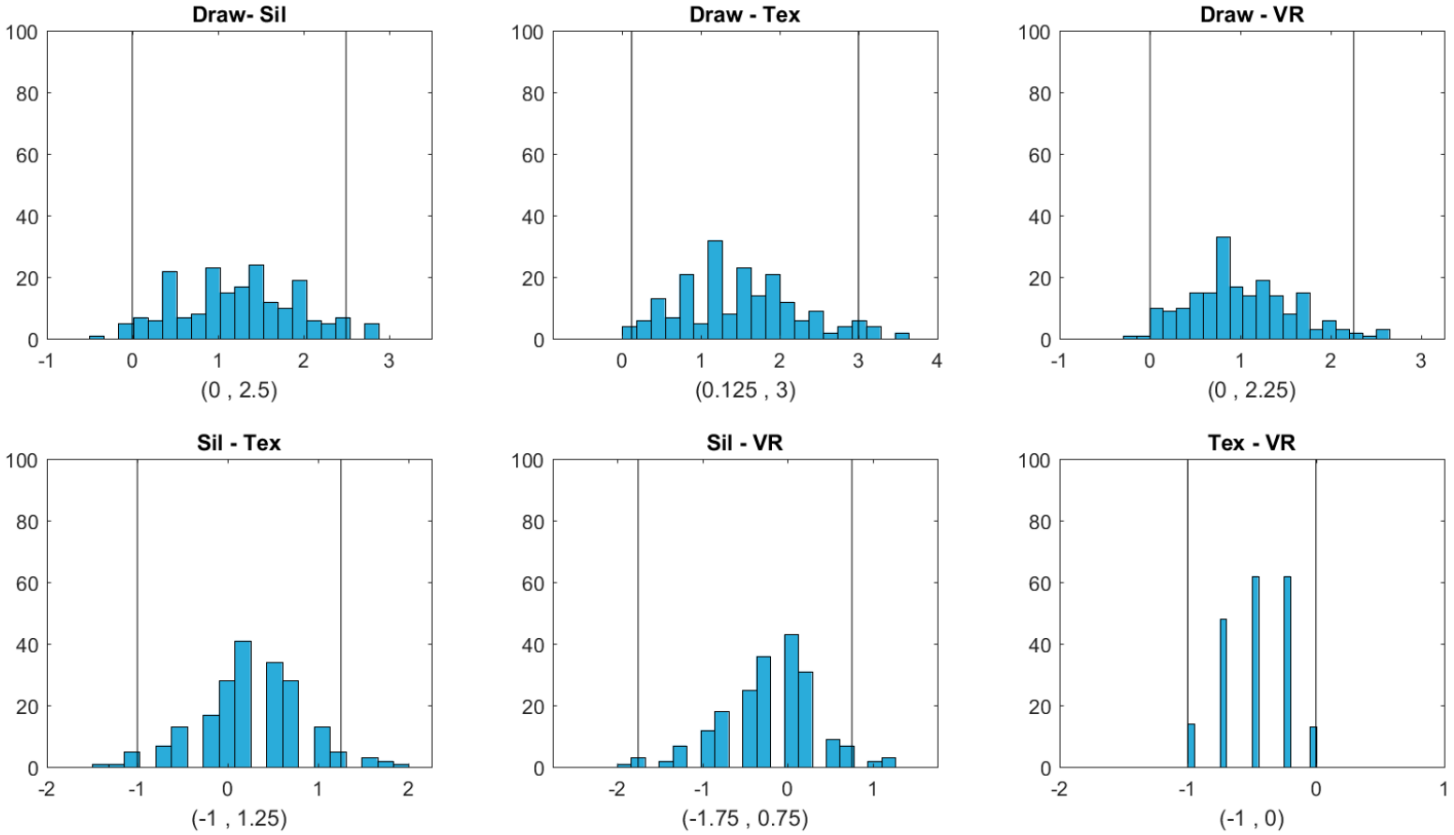
Question 2: Which figure is the size and shape that you would most like to be? Histograms and 95% bootstrap confidence intervals for the pairwise BMI differences between the four image types (Draw = hand drawing, Sil = silhouette, Tex = textured silhouette, and VR = virtual reality) for Question 2 (Which figure is the size and shape that you would most like to be?). The two vertical lines in each plot correspond to the lower and upper bounds of each confidence interval, respectively.

**Figure 6.**
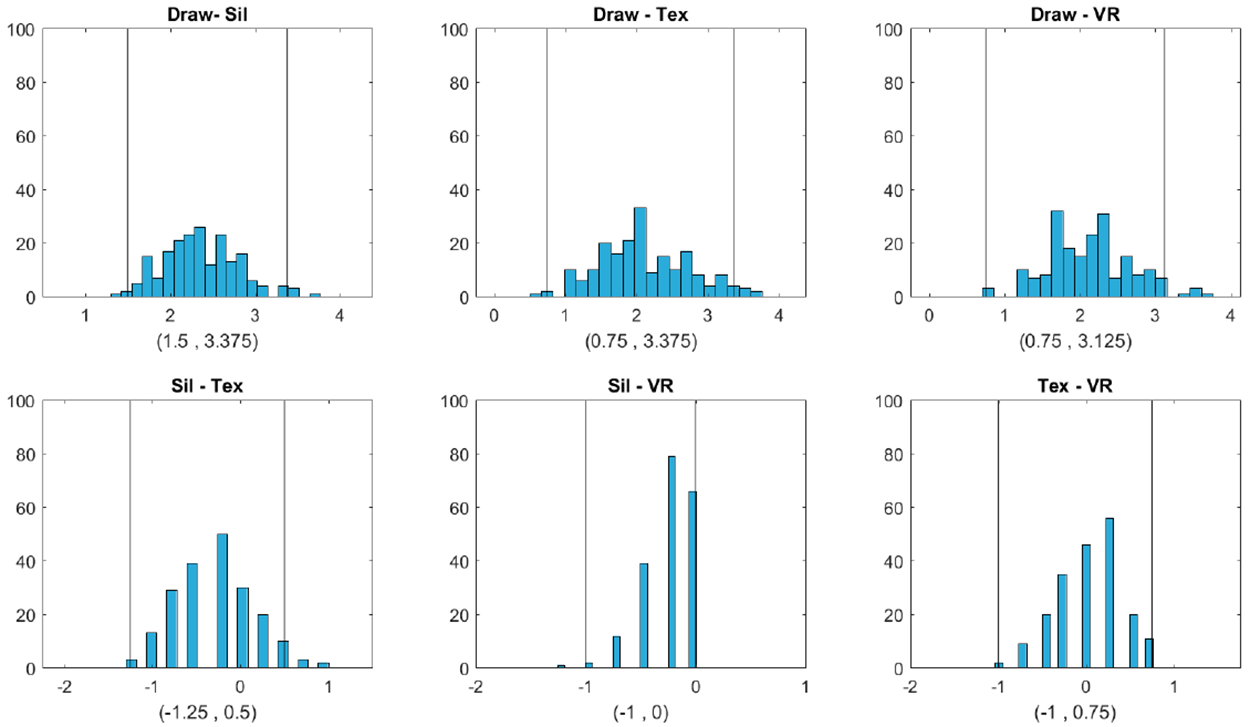
Question 3: Which figure is the size and shape that you feel would be most realistic for you to maintain? Histograms and 95% bootstrap confidence intervals for the pairwise BMI differences between the four image types (Draw = hand drawing, Sil = silhouette, Tex = textured silhouette, and VR = virtual reality) for Question 3 (Which figure is the size and shape that you feel would be most realistic for you to maintain?). The two vertical lines in each plot correspond to the lower and upper bounds of each confidence interval, respectively.

Figures 4-6 show that the BMI values that the subjects selected from the hand-drawn images are often higher than those from the three individualized image types. For Question 1, response BMI values from the hand-drawn condition were significantly higher than those from the silhouette condition (Figure 4a). For Questions 2 and 3, response BMI values from the hand-drawn condition were significantly higher than those from all the other conditions (Figures 5 and 6). In all questions, response BMI values did not differ significantly across the silhouette, 2D textured, and 3D VR conditions.

We further studied the association between these BMI differences and the Body Image QOL Survey [19]. Table 1 provides a list of survey questions of which answers are significantly correlated with the BMI differences observed from the four image types. Table 1 shows that the answers to survey questions S5, S6, S7, S8, S18, and S19 generally have significantly negative correlations with the BMI differences. This implies that, in general, the subjects who had more positive feelings about themselves (e.g., meeting new people, work/school experience, relationship with friends and family members, self-confidence and happiness) were more likely to underestimate their BMI values from the images (Q1), desire lower ideal BMI values than their current ones (Q2), and have stronger confidence in realistically achieving lower BMI values (Q3). The significantly positive correlations regarding the answers to survey questions S13 and S14 with Q2 and Q3 demonstrate that those who were more confident in their abilities to control diet and/or weight tended to be more satisfied with their current BMI values (their desired and estimation of achievable BMI values were closer to their true BMI). The correlations with respect to S19 indicate that the people who believed that they could realistically reach a lower BMI groomed themselves more every day.

## 4. Discussion

This study compared BMI ratings across several different types of body representations in a sample of young adult women. This was an exploratory study that aimed to determine the degree to which personalized realism would affect a woman’s self-perception of her body size. The individualized avatars were created using a new morphing algorithm that yielded biologically realistic variations in avatar BMI. In general, body shape responses using a non-individualized hand-drawn rating scale [5] were often a bit higher than responses using individualized scales; there were few differences between the three conditions containing different amounts of individualized realism, although when subjects rated their current body shape, their responses were most accurate when using the 2D textured and 3D textured VR body representations.

Thaler, Piryankova et al. [21] studied body shape judgments using avatars based on 3D scans of a participant’s body. They manipulated individualized body texture by including avatars with a checkerboard surface texture and avatars with a photo-realistic texture based on the participant’s own body. The checkerboard condition was similar to our silhouette condition, in that it included the participant’s own limb circumferences and dimensions but differed from our silhouettes in that the avatars included shading and texture gradient cues to 3D shape. Their participants were more likely to select avatars with a lower BMI as matching their own body shape than when using avatars having a personalized photo-realistic texture. The data from our silhouette condition revealed a similar pattern—response BMI values significantly underestimated the physical BMI. Thaler, Piryankova et al.’s [21] personalized avatar condition involved viewing a static avatar in an immersive stereoscopic display. Our study included two levels of individualized avatars, one involving a static avatar seen in a 2D helmet-mounted display and a second one involving a 3D rotating avatar in an immersive environment. Our results showed very little difference between these two levels of individualization; both yielded a similar pattern as Thaler et al.’s individualized condition—i.e., response BMI judgments were closer to accurate than those involving avatars without individualized texture. Additionally, in all four conditions, participants chose a lower desired BMI. However, contrary to other studies, this finding was not associated with a negative impact on QOL. This effect may have been mediated by higher confidence in achieving a lower BMI, which was correlated with participants’ ability to control diet and weight. This is consistent with findings that support the importance of self-efficacy in weight management.

The results of our study are also consistent with that of the non-clinical group in the study from Molbert, Thaler, et al. [3], where they found that these women perceived themselves to be smaller when choosing from an individualized avatar figure rating scale, and they also chose a smaller desired body size. These outcomes are also expected from figure rating scales studies, as they coincide with the general social desirability standards of the “thin-ideal” for women in both study samples [38-40]. Molbert’s study utilized personalized avatars to evaluate body size perception in both non-clinical and anorexia nervosa patients by generating morphed images that were ±5–20% BMI of the true image. However, by placing the real image in the middle of the visual options, this increased the potential for contraction bias [13,36] or the bias toward selecting images in the middle of the presented range. The design of the Molbert study was similar to that of a previous study from Piryankova et al. [25] that also placed the true image in the middle of the morphed images. One strength of the current study was that it compared personalized avatars with a generic figure rating scale. Moreover, the morphed images were confined within the range of 18–40 kg·m^−2^ (to resemble the structure of the standard figure rating scale [5]), and thus, the participant’s true image was not always the middle image.

Another aim of Piryankova et al. [25] was to determine the impact that the photo-realistic texture of an avatar would have over an individualized, but not photo-realistic, avatar shape (e.g., checkerboard pattern overlay on the actual avatar). Similar to our findings, they found that adding the photo-realistic texture provided additional visual cues to improve the accuracy of body size self-perception [25]. The Piryankova study also shared a limitation similar to the current study of a small sample size. Given the resource-intensive nature of the current study design and the limitation of available follow-up participants from the original validation study, we utilized bootstrap analysis in an attempt to glean meaningful information from this pilot study. That being said, any inferences from this study are not very generalizable and should be limited to inform future study designs and to further demonstrate the utility of utilizing photo-realistic, individualized figure rating scales [10,21,25]. Moreover, it is recommended that future researchers utilize two strengths highlighted in the current study, which is the BMI-guided morph generation protocol and the fixed BMI range/variable image scrolling selection ability that was utilized to display the images.

In addition to the traditional use of figure rating scales in evaluation of distortions in body image, photo-realistic 3D figure rating scales as developed in the current study could be utilized to demonstrate the beneficial effects of physical activity on changes in body shape. While obese body shapes do not generally meet the societal ideal for attractiveness in many cultures, the same can be said for a female with an athletic physique [38-40]. These perspectives may be changing in Western cultures, but it is clear that physical activity is essential for optimal health and prevention of numerous chronic diseases (e.g., type 2 diabetes, hypertension, breast cancer, etc.) [41-43]. Removing barriers to physical activity, such as lack of social desirability, would be beneficial for both mental and physical health. Future research could utilize these personalized figure rating scales to improve someone’s personal perceptions of what they may look like if they were more physically active.

## 5. Conclusions

This study is based upon a relatively small sample of women largely within the “normal” BMI range, and thus, further research is required to determine the generalizability of these results. However, our results indicate some benefit of individualized texture over and above individualized circumferences and limb lengths (which were present in the silhouette condition) and a tendency for BMI judgments using individualized avatars to be somewhat smaller and more accurate than those using a standardized line drawing scale. Taken together, these results demonstrate that the additional resources required to create individualized avatars to simulate different BMIs do provide some benefit in terms of increasing the accuracy of BMI judgments relative to actual BMI [22]. However, given the similarity in judgments between the 2D textured and 3D textured VR conditions, it appears that an immersive 3D representation that makes more of the body visible does not provide a substantial additional benefit. This could reduce the resources required for administering the individualized scales. It is possible that the sensitivity of figure rating scales could be further improved by more precise changes in the size of the morphed images (e.g., separation by only 1 kg·m^−2^), but future research should investigate the utility and practicality of incorporating such precision into these instruments, as generating these additional images would further increase the resources required. A primary purpose of this exploratory study was to determine the feasibility of generating these individualized, highly realistic avatars in order to improve upon existing figure rating scales. The trends highlighted from this initial study do indicate that it could be worthwhile to create figure rating scales utilizing individualized, textured avatars, but future studies should aim to confirm these findings in a larger, more diverse sample.

## Figures and Tables

**Table 1.** Bootstrap confidence intervals (95%) for the significant correlations between the BMI differences observed from the four image types (Draw = hand drawing, Sil = silhouette, Tex = textured silhouette, and VR = virtual reality) and the Body Image Quality of Life Inventory. A correlation is significant if zero is not contained by the confidence interval.

	Q1: Which Figure Best Reflects How You Think You Look Today?			Q2: Which Figure is the Size and Shape that You Would Most Like to Be?			Q3: Which Figure is the Size and Shape that You Feel Would Be Most Realistic for You to Maintain?		
S5: My experiences when I meet new people	Draw	−0.933	−0.202						
	Sil	−0.966	−0.340				Draw	−0.973	−0.222
	Tex	−0.982	−0.389						
S6: My experiences at work/school	Draw	−0.962	−0.415	Sil	−0.994	−0.027	Draw	−0.988	−0.594
	Sil	−0.992	−0.601	Tex	−0.947	−0.205	Sil	−0.987	−0.468
	Tex	−0.994	−0.696	VR	−0.977	−0.193	Tex	−0.968	−0.639
							VR	−0.962	−0.468
S7: My relationships with friends	Tex	−0.985	−0.540	Tex	−0.842	−0.013	Draw	−0.951	−0.194
							Sil	−0.889	−0.222
							Tex	−0.969	−0.557
							VR	−0.999	−0.501
S8: My relationships with family members	Tex	−0.951	−0.251	Tex	−0.948	−0.058	Draw	−0.978	−0.958
							Tex	−0.958	−0.188
S13: My ability to control what and how much I eat				Draw	0.160	0.947	Sil	0.100	0.976
				VR	0.036	0.994	Tex	0.149	0.982
							VR	0.313	0.993
S14: My ability to control my weight							Sil	0.146	0.978
				VR	0.070	0.998	Tex	0.131	0.983
							VR	0.302	0.989
S17: My daily “grooming” activities							Tex	−0.941	−0.182
							VR	−0.915	−0.123
S18: How confident I feel in my everyday life	Draw	−0.979	−0.204				Draw	−0.985	−0.389
	Sil	−0.915	−0.285				Sil	−0.962	−0.378
	Tex	−0.995	−0.791				Tex	−0.979	−0.631
							VR	−0.948	−0.461
S19: How happy I feel in my everyday life	Sil	−0.880	−0.100						
